# The Root Extract of the Medicinal Plant *Pelargonium sidoides* Is a Potent HIV-1 Attachment Inhibitor

**DOI:** 10.1371/journal.pone.0087487

**Published:** 2014-01-29

**Authors:** Markus Helfer, Herwig Koppensteiner, Martha Schneider, Stephanie Rebensburg, Sara Forcisi, Constanze Müller, Philippe Schmitt-Kopplin, Michael Schindler, Ruth Brack-Werner

**Affiliations:** 1 Institute of Virology, Helmholtz Zentrum München - German Research Center for Environmental Health, Neuherberg, Germany; 2 Research Unit Analytical BioGeoChemistry, Helmholtz Zentrum München - German Research Center for Environmental Health, Neuherberg, Germany; Pasteur Institute of Shanghai, Chinese Academy of Science, China

## Abstract

Global HIV-1 treatment would benefit greatly from safe herbal medicines with scientifically validated novel anti-HIV-1 activities. The root extract from the medicinal plant *Pelargonium sidoides* (PS) is licensed in Germany as the herbal medicine EPs®7630, with numerous clinical trials supporting its safety in humans. Here we provide evidence from multiple cell culture experiments that PS extract displays potent anti-HIV-1 activity. We show that PS extract protects peripheral blood mononuclear cells and macrophages from infection with various X4 and R5 tropic HIV-1 strains, including clinical isolates. Functional studies revealed that the extract from PS has a novel mode-of-action. It interferes directly with viral infectivity and blocks the attachment of HIV-1 particles to target cells, protecting them from virus entry. Analysis of the chemical footprint of anti-HIV activity indicates that HIV-1 inhibition is mediated by multiple polyphenolic compounds with low cytotoxicity and can be separated from other extract components with higher cytotoxicity. Based on our data and its excellent safety profile, we propose that PS extract represents a lead candidate for the development of a scientifically validated herbal medicine for anti-HIV-1 therapy with a mode-of-action different from and complementary to current single-molecule drugs.

## Introduction

As of August 2012, 23 single-molecule drugs were approved for anti-HIV-1 therapy in the USA by the FDA [1]. The continuous need for the development of new therapeutic anti-HIV-1 agents arises from the rapid emergence of viruses resistant to these drugs (reviewed in [2], [3]), the necessity for continuous life-long treatment [4], the challenges of providing antiretroviral treatment in resource-limited settings [5] and the need for novel drugs with fewer adverse effects [6].

Natural products and herbal medicines are a promising source of new therapeutic agents and for the development of complementary and alternative medicines to conventional drug regimens [7]. While medicinal plants have been reported to display anti-HIV-1 activity (reviewed in [8], [9]), herbal preparations are currently not part of conventional therapeutic regimens. Antiviral potencies and modes-of-actions of medicinal plants are poorly understood. Therefore they have been considered mainly as sources for the isolation of single anti-HIV-1 hit molecules by conventional drug-discovery approaches [9], [10]. However, herbal preparations may also contain unique mixtures of molecules that act in concert to display novel bioactivities [11]. Herbal preparations have many potential benefits for anti-HIV therapy, including the complementation of existing drug therapies, improvement of anti-HIV treatment in resource-limited settings and reduction of the risk of emergence of viral resistance. Furthermore, they may display novel modes-of-action, which are different from current single-molecule drugs. Thus it is worthwhile to perform detailed and rigorous experimental investigations to evaluate anti-HIV-1 activities of herbal extracts.

*Pelargonium sidoides* (PS) is an indigenous medicinal plant of South Africa which has been used as a traditional medicine for the treatment of various ailments for over a century [12]. A proprietary extract from PS roots known as EPs®7630 or Umckaloabo® has been evaluated in numerous clinical trials for safety and alleviation of symptoms associated with acute bronchitis and is licensed in Germany as herbal medicine for the treatment of upper respiratory tract infections. PS extract contains numerous different metabolites [13] and has been shown to inhibit viruses associated with respiratory diseases like influenza viruses [14], [15] and herpes virus [16].

The proven safety profile, richness in metabolites and demonstrated activities against various viruses led us to evaluate PS extract for anti-HIV-1 activity. We demonstrate that PS extract potently inhibits infection by HIV-1 strains with different tropisms. Anti-HIV-1 activity of PS extract is based on a new mode-of-action that diminishes infectivity of virus particles and prevents their attachment to host cells. Chemical analysis indicated that anti-HIV activity is mediated by multiple polyphenolic compounds. These results support PS extract as a lead candidate for the development into an herbal medicine with a novel mode of anti-HIV-1 activity.

## Materials and Methods

### Virus production

Virus stocks were produced by HEK293T cells transfected with proviral plasmids and analyzed for infectivity, signal induction, absence of cell toxicity and p24 quantity as described in [17].

The X4- tropic strain HIV-1_LAI_ [18] was produced by transfection with the proviral plasmid pLAI.2, the R5-tropic strain HIV-1_AD8(R5)_ [19] with pNL(AD8), the R5-tropic GFP reporter virus HIV-1 NL4-3 Gag-iGFP [20] with pBR-NL4-3 V92th014.12-IRES-eGFP, the R5-tropic HIV-1ΔEnv_JRFL(R5)_ [21] by co-transfecting pNL4.3ΔEnv and pSVIIIenvJRFL. HIV-1 particles pseudotyped with the VSV-G envelope protein were produced by co-transfecting pSG3ΔEnv and pM2.G (Addgene, MA). Clinical isolates CH077 and STCOr1 were produced by transfection with the appropriate proviral plasmids [22]. P-891 was isolated from serum of an HIV-1 infected individual as described [17]. Non-commercial providers of plasmids are listed in the “Acknowledgements”.

### Cells and cell culture

LC5-CD4 is a HeLa subline engineered for cell-surface expression of CD4 [17]. The HIV-1 reporter cell line LC5-RIC contains a reporter gene for Rev- and Tat-dependent expression of a red fluorescent protein [17]. LC5-RIC-R5 cells were generated by stable transfection of LC5-RIC cells with plasmids containing human CCR5 cDNA in the pMSCVPuro backbone [23]. LC5-RIC, LC5-RIC-R5 and HEK293T (ATCC®-Number CRL-11268) cells were cultured as described [17]. Human peripheral blood mononuclear cells (PBMCs) were isolated from buffy coats by Ficoll (Biochrom AG) gradient centrifugation as described in [24]. Cells were washed and treated with ammonium chloride potassium (ACK) buffer (Gibco), to remove residual platelets and erythrocytes, respectively. PBMCs were stored at -80°C in batches of 1×10^7^ cells pooled from 4 donors in 1 ml VLE-RPMI 1640 medium (Biochrom AG, Berlin) with 20% FCS (Gibco) and 10% DMSO (Sigma Aldrich). Primary human macrophages were isolated from PBMCs as described in [20].

### Reference HIV-1 inhibitors

AZT was purchased from Sigma Aldrich and His-Griffithsin from Biomol GmbH. The following reagents were obtained through the NIH AIDS Research and Reference Reagent Program, Division of AIDS, NIAID, NIH: bicyclam JM-2987 (hydrobromide salt of AMD-3100) [25], [26]; T-20, Fusion Inhibitor from Roche; Efavirenz; His-Griffithsin from Drs. Barry O′Keefe and James McMahon [27].

### Generation of crude PS extracts and isolation of polyphenols

Crude *PS* extract was generated from dried plant roots obtained from Amarelo (www.amarelo24.de). Roots were ground with a ball mill (Minimill Pulverisette 23; Fritsch, Idar-Oberstein). 10 g powdered roots were stirred in 50 ml water (ddH_2_0) for ∼2h at room temperature (RT). Boiling, while not necessary, did not affect anti-HIV activity. The extract was cleared by centrifugation (4000 g). Aliquots of the extract were evaporated in an Eppendorf Vacuum Concentrator, the dry mass weighed and ddH_2_0 added to generate extract stock solutions with 10 mg dry mass per ml. All PS extract stock solutions were sterilized by filtration (0.45 µM syringe-filter; Merck Millipore Ltd) and stored at -20°C until use.

#### Enrichment of *PS*-derived polyphenols (PSPP)

2.5 g of polyvinylpyrrolidone (PVPP; Sigma-Aldrich) was added to 50 ml of PS extract stock solution (10 mg/ml), vortexed, and polyphenols allowed to adsorb to PVPP at RT for 15 min before centrifugation at 4000 g. The supernatant represented the polyphenol-depleted fraction and the pellet the polyphenol-enriched fraction. The pellet was washed 3 times with 25 ml ddH_2_0 and polyphenols eluted by washing 3 times with 25 ml 0.5 N NaOH. The pooled eluates were adjusted to pH 7 with 2 N HCl. The polyphenol-depleted and polyphenol-enriched fractions were purified by solid phase extraction (SPE) (Agilent Bond Elut C18). SPE-purified eluates were evaporated and dissolved in ddH_2_0 to generate stock solutions with 10 mg per ml.

### Assays

Assays for inhibition of HIV-1 infection of LC5-RIC cells and primary human cells are described in Materials and Methods S1.

HIV-1-cell attachment assays were performed with the HIV-1 NL4-3 Gag-iGFP reporter virus and LC5-RIC-R5 cells in the presence of the HIV-1 fusion inhibitor T-20 (50 nM). LC5-RIC-R5 cells (2×10^5^) were seeded onto 24×24 mm cover slips placed in 6-well plates. 24 hours later, the culture medium was replaced by fresh medium containing inhibitor compounds (100 µg/ml PS-extract or polyphenol enriched PS-extract, or 5 nM Griffithsin reference compound) and virus inoculum (8.5 pg p24/cell). After 4 h incubation at 37°C, the cells were washed once with PBS and cell membranes stained with DiD (Invitrogen) according to the manufacturer's protocol. Cells were then fixed in 2% paraformaldehyde (PFA) in PBS at room-temperature for 10 minutes and treated with DAPI-staining solution for 5 minutes at room temperature for staining of cellular DNA. The cells were washed, coverslips fixed onto glass-slides with Moviol and dried in the dark overnight. Samples were analyzed by fluorescence microscopy (Nikon TiE equipped with Perkin Elmer UltraView Vox System). Exposure-times: GFP: 100 ms, DAPI: various, DiD: various, Brightfield: various. Counting of virus particles (GFP spots defined as all pixels within a radius of 1 µm around the brightest spot, intensity threshold 755) was performed using Volocity 6.2.1-software (Perkin Elmer). DiD/DAPI-positive cells were counted manually.

Cell viability was analyzed by MTT (3-(4,5-Dimethylthiazol-2-yl)-2,5-diphenyltetrazolium bromide) assay as described in [17].

### Quantification of HIV-1 DNA and RNA levels

HIV-1 DNA copy numbers and viral RNA were determined by quantitative PCR as described in Materials and Methods S1.

**Chromatographic Separation of PS Extract** was performed by UPLC (Ultra Performance Liquid Chromatography) of PS extract purified by solid phase extraction (for details see Materials and Methods S1).

**Ultrahigh Resolution Mass Spectrometry** was performed by ICR/FTMS (Ion Cyclotron Resonance/Fourier Transformation Mass Spectrometry). Details, including description of methods for evaluation of complex mass data, are provided in Materials and Methods S1.

### Statistical Analysis

EC_50_/CC_50_ values were calculated with GraphPad Prism v5, using the equation for sigmoidal dose-response with variable slope. Statistical significances were determined by one-way Anova with Bonferroni post-hoc test. **** p<0.0001.

## Results

### Anti-HIV-1 activity of PS root extract

**Figure 1:**
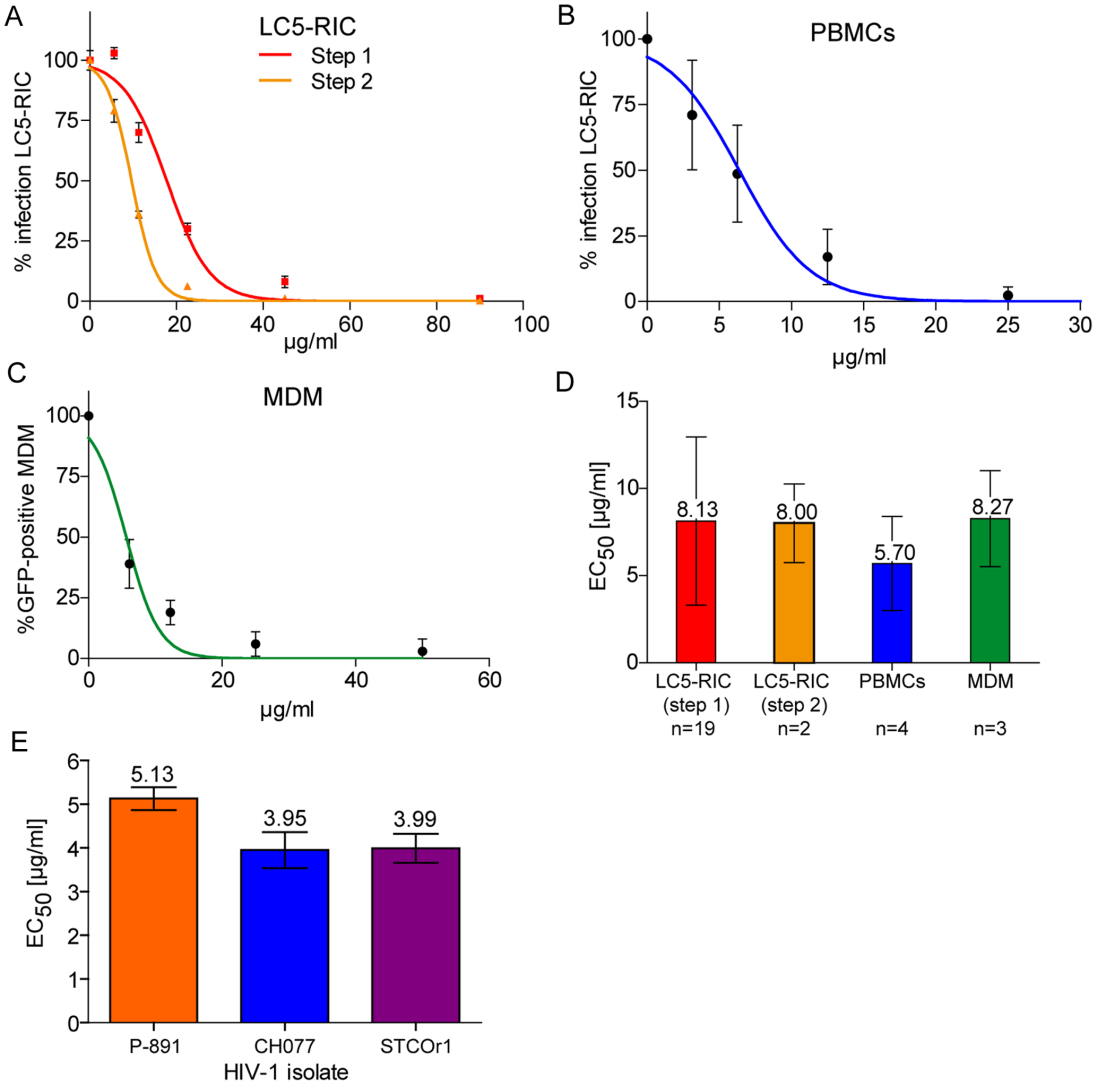
Evaluation of anti-HIV-1 activity of *Pelargonium sidoides* (PS) aqueous root extract with various HIV-1 target cells and HIV-1 isolates. Panels A-C show representative results for dose-dependent inhibition of HIV-1 infection of LC5-RIC indicator cells, human peripheral blood mononuclear cells (PBMC) and human monocyte-derived macrophages (MDM) by PS extract. Data for infection of PS-treated cultures are expressed in percentage of values measured for untreated cultures ( = 100% infection). The graph in D shows the EC_50_ values calculated from multiple independent infection experiments (n = number of independent infection experiments) for each cell type. Panel E shows HIV-1 inhibitory activity (EC_50_) of PS extract against clinical HIV-1 isolates. *A*, HIV-1 reporter cells (LC5-RIC) were exposed to HIV-1_LAI_ and serial dilutions of PS extract and infection assayed by measuring reporter gene expression (step 1) and production of infectious virus (step 2). Mean values and standard deviation of the mean are indicated for each extract dilution. *B*, IL-2 stimulated PBMCs were exposed to HIV-1_LAI_ and different concentrations of PS extract and levels of infectious virus in culture supernatants of PBMCs assayed with LC5-RIC cells. Results are shown for 4 independent infection experiments using PBMCs that were pooled from 4 different donors, with each extract concentration assayed in triplicate in each experiment. *C*, MDMs were exposed to the HIV-1 reporter virus R5 HIV-1NL4-3 IRES-eGFP that coexpresses Nef and EGFP from a bicistronic mRNA via an IRES element. Three days later, proportions (%) of GFP positive cells were determined by flow cytometry. Data were normalized to values for untreated cells. Results are shown for three independent infection experiments with MDMs from different donors. *D*, The graph shows EC_50_ values (effective concentration for half-maximal inhibition) for each cell type, calculated from the compiled results of multiple independent infection experiments. n = number of independent infection experiments with triplicate analysis of each extract concentration. Mean values and standard deviations of the mean are indicated for each cell type. *E*, HIV-1 reporter cells (LC5-RIC-R5) were exposed to the indicated clinical HIV-1 isolates and serial dilutions of PS extract in triplicate wells and reporter gene expression was measured 5 days post infection. Mean values and standard deviations of the mean are indicated for each HIV-1 isolate.

Initial testing of PS-root extract for anti-HIV-1 activity with EASY-HIT technology [17] revealed dose-dependent inhibition of HIV-1 infection for all extract preparations examined, including the commercial herbal medicine EPs®7630 (Figure S1). The EASY-HIT assay gauges antiviral effects by measuring infection parameters associated with two temporally distinct steps of the HIV-1 replication cycle (i.e. expression of early viral proteins Tat and Rev  =  step 1 and release of infectious virions  =  step 2, resp.) with an HIV-1 reporter cell line [17]. Multiple tests showed that PS extract was similarly effective at reducing infection parameters at both steps, with half-maximal inhibition (EC_50_) at 8.13 µg/ml (±4.82; n = 19) for step 1 and 8.00 µg/ml (±2.26; n = 2) for step 2 (Figure 1). This indicates that PS extract interferes with HIV-1 replication at an early step of the HIV-1 replication cycle [17].

To determine anti-HIV activity of PS extract with primary HIV-1 target cells, we used *ex vivo* cultures of human peripheral blood mononuclear cells (PBMC) and monocyte-derived macrophages (MDM). Both primary HIV-1 target cell types yielded EC_50_ values comparable to those measured for the reporter cells in the EASY-HIT system (Figure 1). Inhibition of infection of PBMCs by PS extract was determined by measuring infectious virus titers of supernatants of cultures exposed to HIV-1_LAI_ and different concentrations of PS extract (EC_50_ = 5.70±2.7 µg/ml; n = 4). PS-mediated inhibition of infection of MDMs was analyzed with an R5 reporter virus that coexpresses Nef and EGFP from a bicistronic mRNA (R5 HIV-1NL4-3 IRES-eGFP [20]) and analyzing proportions of EGFP-positive cells by flow cytometry (EC_50_ = 8.27±2.75 µg/ml; n = 3).

We also investigated whether PS extracts can inhibit infection by clinical HIV-1 isolates. Clinical isolates were derived from serum of a chronically infected individual (P-891; [17]) and from blood of acutely (CH077) or chronically (STCOr1) infected individuals [22]. As shown in Figure 1*E*, PS extract was active against all clinical isolates investigated, yielding EC_50_ values below 6 µg/ml.

In all assays evaluating PS-mediated inhibition of HIV-1 infection, viability of cells exceeded 80%, confirming that inhibition of infection was not caused by PS-induced cytotoxicity.

Together, these results demonstrate that PS extract inhibits HIV-1 infection of various HIV-1 target cell types, including primary cells, and acts against various HIV-1 isolates, including clinical HIV-1 isolates.

### PS extract interferes with HIV-1 entry into the host cell

**Figure 2:**
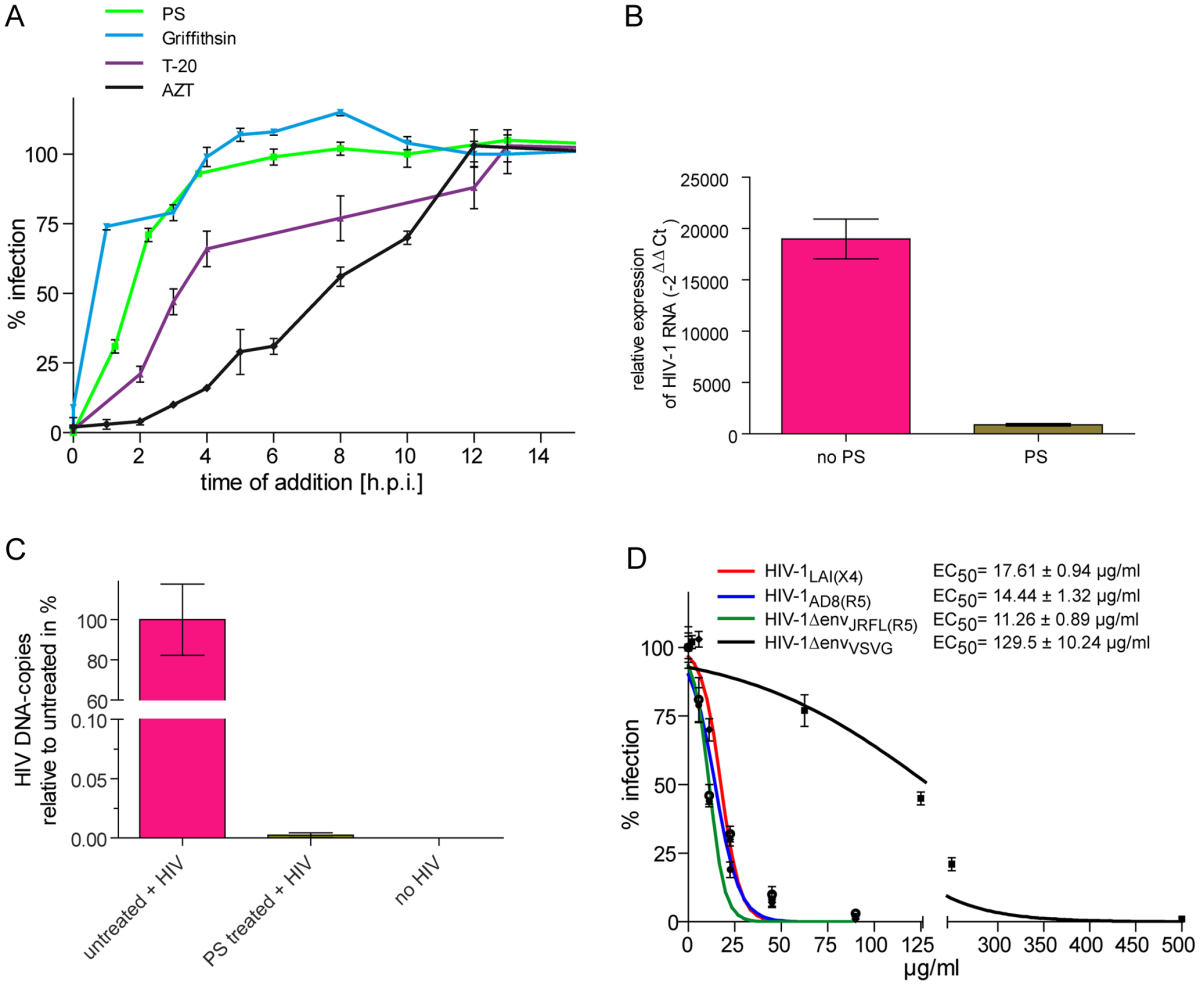
PS extracts inhibit entry of HIV-1 into the host cell. *A*, Comparison of time-of-addition (TOA) profiles of PS extract and reference compounds that inhibit HIV-1 entry (i.e. Griffithsin and T-20) and reverse transcription (AZT), respectively. For TOA assays, virus preparations (HIV-1_LAI_) were added to LC5-RIC cells and HIV-1 inhibitors added either simultaneously with the virus (time point 0) or at later time points (h.p.i.  =  hours post infection) at concentrations yielding maximum inhibition (i.e. >5× EC_50_). Cultures were assayed for reporter expression 48 hours after virus addition and fluorescent signal intensities normalized to those of cultures exposed to virus in the absence of inhibitors. Each time point was measured in triplicate. Mean values and standard deviation of the mean are indicated for each time point. *B*, Reduction of input HIV-1 viral RNA levels by treatment with PS-extract. LC5-CD4 cells were exposed to HIV-1_LA1_ and Efavirenz in the presence or absence of PS extract (50 µg/ml) in triplicate samples. After 4 hours exposure time, RNA was isolated from each sample, cDNA prepared and real-time PCR performed with HIV-1 specific primers to quantify viral RNA levels. HIV-1 transcript levels were normalized to RPII. Relative expression was calculated by the −2^−ΔΔCT^ method. The data is expressed as fold expression of HIV-1 RNA in HIV-1 exposed/treated samples compared to unexposed/untreated samples. Columns indicate mean values and error bars the standard deviations of the means. *C*, Reduction of HIV-1 DNA loads in HIV-1-exposed PBMCs by treatment with PS extract. HIV-1 DNA levels were determined by real-time PCR in peripheral blood derived mononuclear cells treated with PS extract during exposure to HIV-1 and in untreated infected cells (analysed in triplicate samples). Columns indicate mean values and error bars the standard deviations of the means. *D*, Influence of envelope proteins on the inhibition of virus infection by PS extract. LC5-RIC cells were exposed to HIV-1 variants with different HIV-1 envelope proteins representing different virus tropisms (HIV-1_LAI_: X4-tropic; HIV-1-AD-8 and HIV-1ΔEnv_JRFL(R5)_: R5-tropic) or HIV-1 based virus particles pseudotyped with an envelope protein (G-protein) from a heterologous virus (*Vesicular Stomatitis Virus*; HIV-1ΔEnv_VSVG_) in the presence of different concentrations of PS extract. Cultures were assayed for reporter expression 48 hours after virus addition and fluorescent signal intensities normalized to those of cultures exposed to virus without inhibitors. Mean values of triplicate wells and standard deviation of the mean are indicated for each extract concentration.

To narrow down the stage of the HIV-1 replication cycle at which PS extract inhibits HIV-1 replication, we first performed time-of-addition (TOA) assays with PS extract [17], [28]. The HIV-1 entry inhibitors Griffithsin [27] and T-20 [29] and the reverse transcription inhibitor AZT [30] were used as reference compounds. The TOA profiles indicate that PS extract acts at a very early stage of HIV-1 replication which precedes reverse transcription (Figure 2*A*). For further confirmation, we investigated whether treatment with PS extract reduced levels of input viral RNA. Experiments were performed in the presence of reverse transcriptase inhibitor (Efavirenz) to maximize accumulation of input viral RNA. RNA was isolated from cells exposed to the virus +/- PS extract for 4 hours and levels of input RNA quantified by RT-PCR using HIV-1 specific primers. As shown in Figure 2*B*, PS treatment led to a strong reduction of input viral RNA levels in virus-exposed cells, compared to levels of HIV-1 RNA in cells exposed to the virus without PS treatment. As expected, PS treatment also strongly diminished levels of HIV-1 DNA in cells exposed to the virus without Efavirenz (Figure 2*C*). Together, the facts that PS extract behaves like validated entry inhibitors in TOA experiments and the dramatic reduction of levels of input HIV-1 RNA and HIV-1 DNA in virus-exposed cells caused by treatment with PS extract indicate that PS blocks HIV-1 entry into host cells.

Entry is mediated by the HIV-1 envelope protein. Therefore we investigated the influence of viral envelope proteins on the antiviral activity of PS by comparing inhibitory effects of PS extract on infection by HIV-1 particles pseudotyped with different envelope proteins. Virions carrying the vesicular stomatitis virus G-envelope protein on their surface were on average 10-fold less sensitive to inhibition by PS extract than HIV-1 with native envelope proteins (Figure 2*D*). In contrast, PS extract was similarly efficient at inhibiting infection of virus particles with different HIV-1 envelope proteins (i.e. X4 (LAI) and R5 (AD8 and JRFL)). These results demonstrate that PS extract inhibits HIV-1 entry by interfering with the function of the envelope proteins independent of their coreceptor tropism.

**Figure 3:**
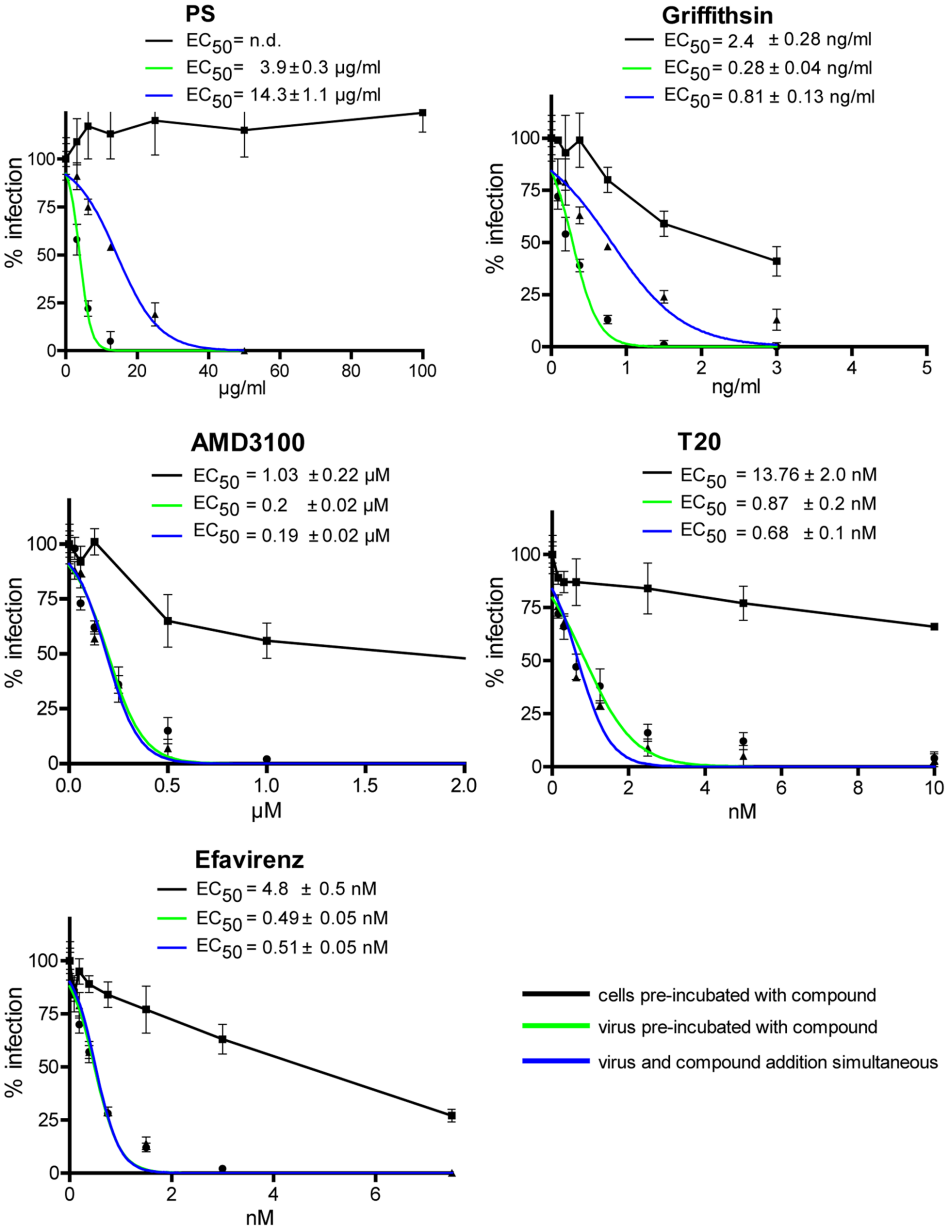
Effects of pre-incubation of virus preparations or target cells with PS extracts or reference compounds on inhibition of HIV-1 infection. Inhibition of HIV-1 infection by PS extract was enhanced by pre-incubation of virus preparations with PS extract, compared to inhibition of infection without virus pre-incubation. HIV-1 target cells pre-incubated with PS extract prior to infection did not retain HIV-1 inhibitory activity. Virus pre-incubation (green curves): Inhibitory activity of PS extract was assayed with HIV-1_LAI_ virus preparations pre-incubated with different concentrations of PS extract for 4 hours before adding to LC5-RIC cells. Target cell pre-incubation (black curves): LC5-RIC cells were incubated with different concentrations of inhibitors and subsequently exposed to HIV-1_LAI_ in fresh medium in the absence of inhibitors. No pre-incubation (blue curves): Virus and inhibitors were added simultaneously to LC5-RIC cells. The entry inhibitors Griffithsin, AMD3100 and T20 and the reverse transcriptase inhibitor Efavirenz were analyzed as reference compounds. Mean values of triplicate wells and standard deviation of the mean are indicated for each extract concentration.

Since PS extract perturbs functionality of the HIV-1 envelope, we investigated whether PS extract interferes with infectivity of HIV-1 particles. Virus preparations were pre-incubated with PS extract without target cells prior to assaying their infectivity. Pre-incubation assays were also performed with the reference compounds Griffithsin, the CXCR4 antagonist AMD3100 [31], the fusion inhibitor T-20 and the reverse transcriptase inhibitor Efavirenz [32]. As shown in Figure 3, virus pre-incubation with PS extract resulted in stronger inhibition of infection (PS panel, green curve, EC_50_ 3.9±0.3 µg/ml) than simultaneous addition of virus and PS extract to LC5-RIC cells (PS panel, blue curve, EC_50_ 14.3±1.1 µg/ml). Virus pre-incubation also enhanced the inhibitory activity of Griffithsin, but not of the other reference compounds (other panels, green and blue curves).

Next we tested whether anti-HIV-1 activity of PS extract involves association of extract ingredients with target cells LC5-RIC target cells were pre-incubated with PS extract or the reference inhibitors for 4 hours, and subsequently exposed to HIV-1_LAI_ virus in the absence of inhibitor. While PS extract displayed no antiviral activity under these conditions (Figure 3, PS panel, black curve), all reference inhibitors showed measurable anti-HIV-1 activities (other panels, black curves), although the compounds were removed and cells were washed after the 4-hour pre-incubation period.

Together these results show that PS extract is an HIV-1 entry inhibitor that directly diminishes the infectivity of viral particles.

### PS extract inhibits attachment of HIV-1 to host cells

**Figure 4:**
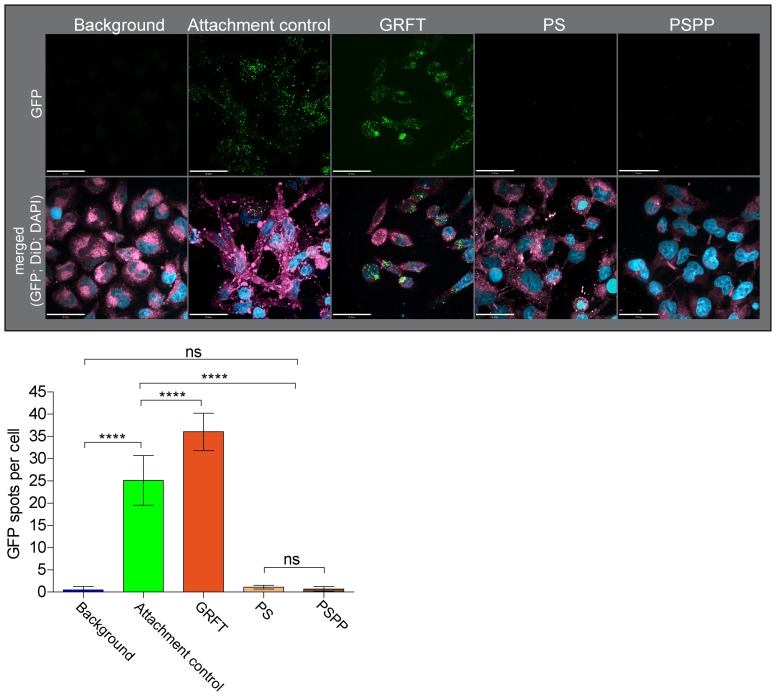
PS extracts inhibit attachment of virus particles to host cells. The effects of PS extract (PS), polyphenols enriched from PS extract (PSPP) by adsorption to polyvinylpolypyrrolidone and the reference compound Griffithsin (GRFT) on virus attachment were determined by fluorescent imaging of virus particles associated with host cells. Controls consisted of cells without virus inoculum (background) and cells exposed to virus particles in the absence of inhibitory compounds (attachment control). LC5-RIC-R5 cells were exposed to fluorescently tagged R5 virus particles (R5 HIV-1 NL4-3 Gag-iGFP) in the presence of the fusion inhibitor T20. Cells were stained with DiD (membrane stain; pink) and DAPI (DNA stain; blue). *A*, Representative images are shown for each sample. Scale bars: 35 µm. *B*, Quantitative analysis of cell-associated fluorescent signals. Quantification of cell-associated GFP signals was performed with Volocity 6.2.1 imaging software. Cells were identified manually. 160-180 cells from at least 5 images were analyzed for each sample. Mean values and standard deviations are shown. Statistical significances were determined by one-way Anova with Bonferroni post-hoc test. **** p<0.0001.

PS extract might target a pre- or post binding step to diminish HIV-1 infectivity and inhibit entry. We thus investigated whether PS extract affects the attachment of HIV-1 to target cells. Assays were performed with GFP-labeled infectious R5 virus particles (R5 HIV-1 NL4-3 Gag-iGFP; [20]) and LC5-RIC-R5 cells. Spinning disc confocal fluorescence microscopy was used to visualize and quantify GFP-labeled HIV-1 particles associated with target cells. All assays were performed in the presence of the fusion inhibitor T-20 to promote accumulation of cell surface bound HIV-1. Tests were performed with HIV-1 inhibitor solutions validated for anti-HIV-1 activity (EC_50_ PS: 7.9±0.8 µg/ml; GRFT: 0.34±0.04 nM). The results are shown in Figure 4. As expected, GFP-labeled HIV-1 particles efficiently attached to the cells in the absence of inhibitor (attachment control, 25.1±5.6 GFP-spots per cell). In contrast, PS extract completely blocked cellular HIV-1 attachment, yielding similarly low numbers of GFP signals per cell as the background control (PS, 1.1±0.4; background, 0.5±0.7). Treatment with Griffithsin resulted in a small although significant increase in the number of cell-associated virus particles (36±4.2 GFP-spots per cell), which is in line with previous evidence for the enhancement of virus binding to the CD4 receptor by Griffithsin [33]. Thus, PS extract prevents HIV-1 attachment to target cells, which is a novel mode of entry inhibition.

### Anti-HIV-1 activity of PS extract is mediated by polyphenol compounds

The high complexity and diversity of the metabolic pool of the PS plant [13] raises the possibility that anti-HIV-1 activity of PS extract may be exerted by multiple compounds. For chemical characterization of anti-HIV-1 activity, PS extract was separated by UPLC-chromatography and anti-HIV-1 activity of fractions determined with the EASY-HIT system. Chemical structures within the fractions were identified by ICR/FTMS (Ion Cyclotron Resonance/Fourier Transformation Mass Spectrometry). This technology allows to distinguish between several thousands of ions with extremely high mass accuracy [34] and enables prediction of the molecular formula of detected compounds and their putative annotation [35].

**Figure 5:**
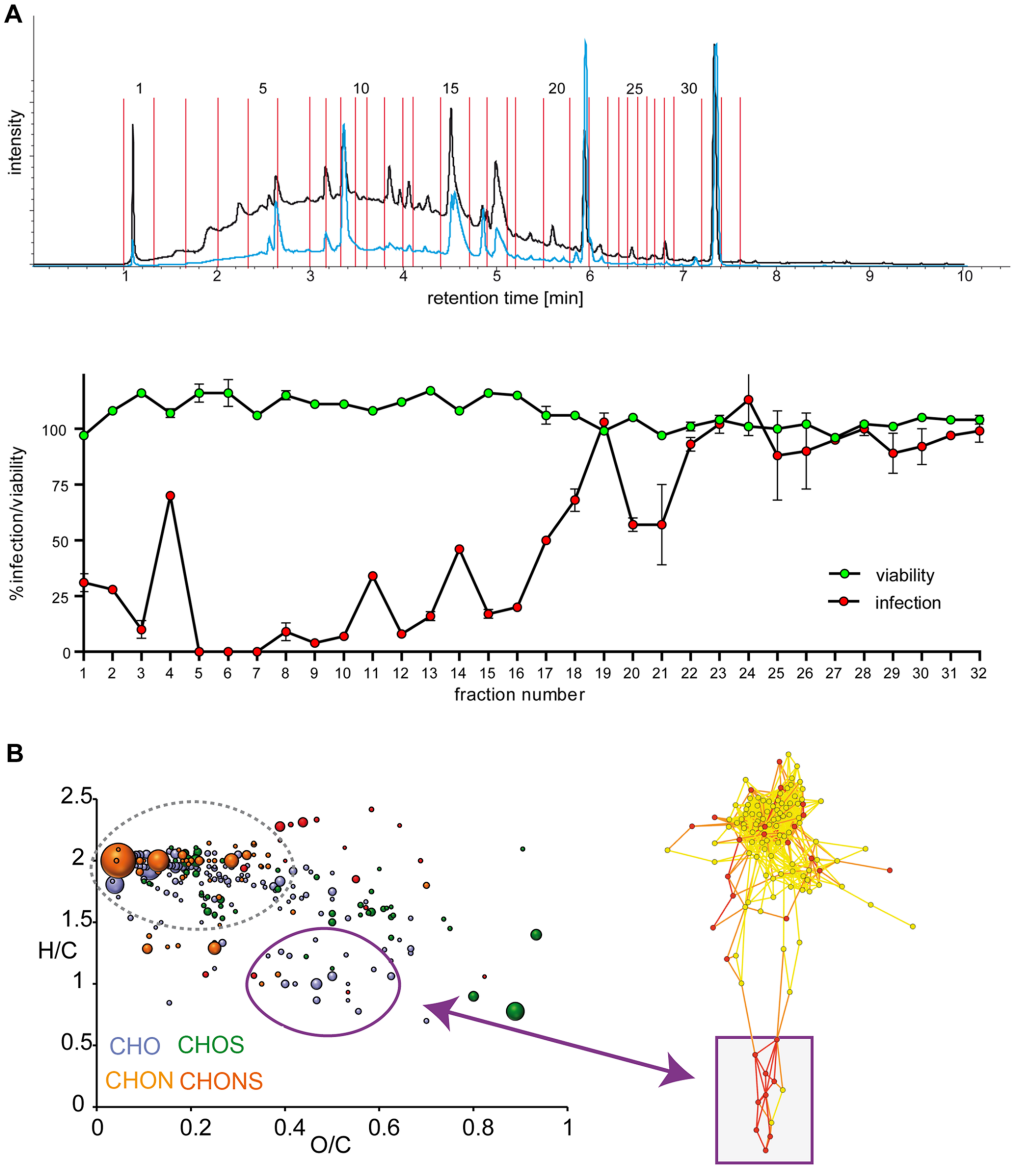
Chemical analysis of anti-HIV activity of PS extract. PS extract was purified by solid phase extraction and separated by UPLC-chromatography. Fractions were analyzed for anti-HIV activity with the EASY-HIT assay and for effects on cell viability by MTT assay. Chemical structures in fractions were analyzed by a combination of ICR/FTMS (Ion Cyclotron Resonance/Fourier Transformation Mass Spectrometry), structural data-mining, statistical analysis and mass network analysis. *A*, Profiling of anti-HIV activity shows inhibitory activity in multiple UPLC-fractions. The UV-chromatogram (top) displays the signal intensities at 289 (black graph) and 330 (blue graph) nm used for peak-oriented identification of PS fractions. The profile below the UV-chromatogram shows a plot of the anti-HIV-1-activity (red data points) and cell viability (green data points) of the corresponding UPLC fractions. *B*, Chemical diversity of anti-HIV activity. The van Krevelen diagram at the left shows the chemical diversity of compounds discriminating fractions with strong anti-HIV-1 activity (3, 5-10, 12) from non-active fractions (19, 22-32). Discriminatory compounds included groups of polyphenolic compounds like flavonoids and leuco-anthocyanidins (encircled in violet) as well as aliphatic oxygen poor, hydrogen rich lipid compounds (encircled in grey). The graph at the right shows mass networks in highly active fractions (i.e. fractions 5-10, 12, 13, 15, 16; red) versus poorly active fractions (1, 2, 4, 11, 14, 17, 19-21, yellow). The networks include a cluster of masses predominantly associated with high anti-HIV activity (framed in violet) that map to the group of polyphenolic compounds in the van Krevelen diagram (violet arrow).

Several fractions dispersed throughout the chromatogram displayed anti-HIV-1 activity (Figure 5*A*). This indicates that multiple chemical compounds in the PS extract have anti-HIV-1 activity. ICR/FTMS analysis combined with structural data-mining, statistical analysis and mass network analysis revealed that fractions with anti-HIV-1 activity were selectively enriched for various aromatic and oxygen rich compounds (Figure 5*B*). These were annotated as polyphenolic compounds, mainly belonging to the classes of flavonoids and leuco-anthocyanidins.

To investigate involvement of polyphenols in anti-HIV activity of PS extract, we used adsorption of polyphenols to polyvinylpolypyrrolidone [36] to produce a PS-derived polyphenol fraction (PSPP). Specific enrichment of polyphenols in this fraction was confirmed by ICR/FTMS (Figure S2).

**Figure 6:**
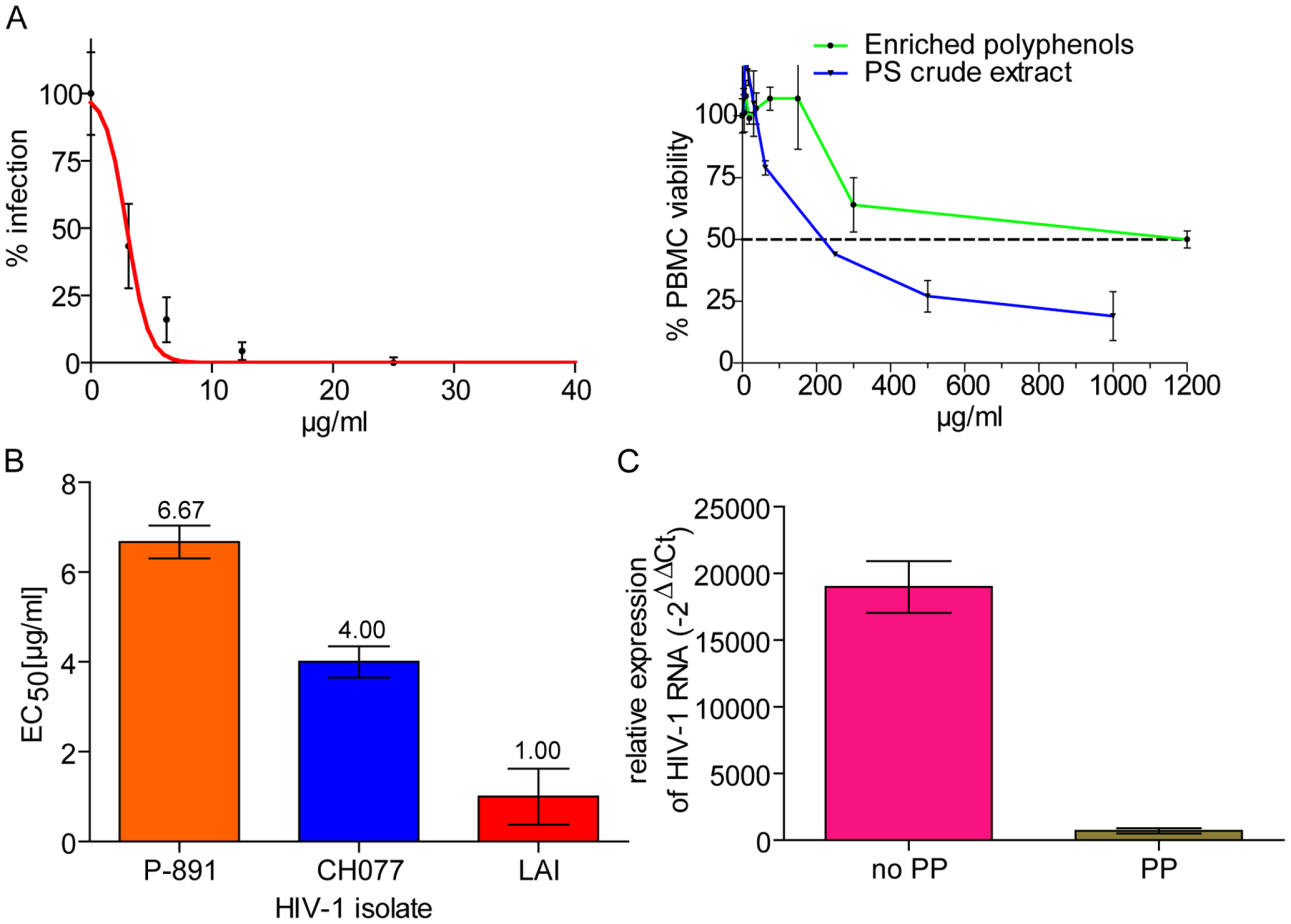
PS-derived polyphenols (PSPP) show potent anti-HIV-1 activity with low cytotoxicity. Polyphenols were isolated from PS extract by adsorption to polyvinylpolypyrrolidone. *A*, Effects of PSPP treatment on HIV-1 infection and viability of PBMCs. The left panel shows dose-dependent inhibition of infection of PBMCs with HIV-1_LAI_. The data represents results from 3 independent infection experiments in which quadruplicate wells were assayed for each extract concentration. Mean values and standard deviations of the means are indicated. The EC_50_ value calculated from these data is 2.98±0.2. The right panel shows the effects of enriched polyphenols and the crude PS extract on the viability of PBMCs. PBMCs were exposed to different concentrations of PSPP or the crude PS extract and effects on cell viability measured by MTT assay. Mean values and standard deviations of the means are indicated for each concentration (triplicate samples). *B*, Anti-HIV-1 activity of PSPP against different HIV-1 isolates. For further details see legend to Fig. 1E. *C*, Reduction of input viral RNA levels by PSPP treatment. For details see legend to Figure 2*B*.

Analysis of anti-HIV-1 activity of the PSPP fraction showed that it also inhibited HIV-1 infection (Figure 6*A, B*). Anti-HIV-1 activity of the PSPP extract was confirmed with primary target cells (PBMCs) and HIV-1 reporter cells (LC5-RIC) and for different HIV-1 isolates, including clinical isolates. The EC_50_ values for PSPP-mediated HIV-1 inhibition were <7 µg/ml, and were comparable to the EC_50_ values measured for the crude PS extract (Figure 1). In contrast, anti-HIV-1 activity of the polyphenol-depleted fraction was severely diminished (EC_50_ ∼200 µg/ml; not shown). Strikingly, the PSPP fraction was much less cytotoxic for PBMC [50% cytotoxic concentration (CC_50_)≥1200 µg/ml] than the crude PS extract (CC_50_≤250 µg/ml) (Figure 6*A*). This resulted in a strongly improved therapeutic index (CC_50_/EC_50_) for PSPP (324), which is about 9-fold higher than for crude PS extract (∼35). PSPP treatment also inhibited attachment of virus particles to cells (Figure 4) and dramatically reduced input viral RNA levels (Figure 6*C*) indicating that PSPP inhibits HIV-1 by the same mechanism of action as crude PS extract.

In sum, the antiviral activity of PS extract can be attributed to polyphenolic secondary metabolites belonging to the classes of flavonoids and leuco-anthocyanidins. The potent anti-HIV-1 activity and reduced cytotoxicity of the PSPP fraction indicate that enrichment of polyphenols from PS root extracts eliminates cytotoxic molecules, while retaining HIV-1 inhibitors.

## Discussion

This study demonstrates that PS root extract, including the commercial herbal medicine EPs®7630, is a highly reliable source of robust anti-HIV-1 activity. PS extract prevents attachment of virus particles to host cells and hence HIV-1 entry. Our results suggest that HIV-1 inhibitory molecules in PS extracts target HIV-1 envelope proteins, since virus particles bearing the heterologous VSV-G protein instead of HIV-1 proteins in their envelopes are much less sensitive to inhibition by PS extract. The mode-of-action of PS extract exhibits a combination of features that set it apart from approved anti-HIV-1 drugs and from investigational entry inhibitors including lectins [37], [38], polyanionic compounds [39], [40] and synthetic anti-lipopolysaccharide peptides (SALPs) [41], of which several are under development as microbicides and drug candidates. For one, PS extract prevents attachment of virus particles to host cells. In contrast, the lectin Griffithsin, which was assayed as reference compound, did not inhibit HIV-1 attachment and therefore blocks entry at a later stage. Other plant derived HIV-1 entry inhibitors epigallocatechin gallate (EGCg) and theaflavin also act after attachment [42], [43], [44] and in this respect are similar to Griffithsin but different from PS extract. Second, PS exerts potent anti-HIV-1 activity independent of the viral coreceptor tropism. This is in contrast to polyanions that mainly target CXCR4-using HIV-1 strains [45], and to compounds that act by blocking single HIV-1 cell surface co-receptors (e.g. AMD3100 [31]) or CCR5 (e.g. Maraviroc; [46]). Third, PS extract directly interferes with the infectivity of HIV-1 particles before they interact with the host cell and thus has virucidal activity. This is in contrast to entry inhibitors that bind to molecules on the surface of host cells (e.g. SALPs, Maraviroc and Griffithsin [47]) or that interfere with entry processes that take place after the virus has bound to the host cell (T20).

Interestingly, PS extract was also reported to display virucidal activity against herpesvirus [16], but not against influenza virus [15]. This indicates that PS inhibits HIV-1 and herpesviruses by mechanisms different from those involved in inhibition of influenzavirus and suggests that PS extract contains multiple antiviral compounds with different activities.

In addition to the novel mode-of-action, PS extract has several other characteristics that highlight it as an attractive candidate for HIV-1 preventive and therapeutic strategies. We demonstrate that anti-HIV activity of PS extract is exerted by the concerted action of a unique combination of polyphenolic antiviral ingredients. Conceivably, this may reduce the risk of the emergence of drug-resistant viruses, compared to treatment with single-molecule drugs.

Furthermore, safety of PS extract was demonstrated in at least 18 clinical trials, involving treatment of children and adults with upper respiratory tract diseases with EPs®7630 over several weeks [12], [48]. We show that enrichment of polyphenols by adsorption to polyvinylpyrrolidone further reduces cytotoxicity for PBMCs, improving the therapeutic index of PS-mediated antiviral activity. Clinical safety of PS extracts and the compelling evidence for *in vitro* anti-HIV-1 activity presented in our study encourages future testing of PS extract and PS-derived polyphenol-enriched formulations in HIV-1 infected individuals.

Because PS extract inhibits HIV-1 entry by a novel mode-of-action, it may complement the activity of current microbicide candidates and thus improve prevention of HIV-1 transmission. For anti-HIV-1 therapy, PS extract may supplement first-line treatment regimens, which so far do not include entry inhibitors. Furthermore, PS extract may help decrease virus burden during interruption of conventional drug treatment. Another remarkable feature of the anti-HIV-1 activity of PS extract is its robustness against boiling, drying and extreme pH conditions. Thus we show that PS-extract can be easily produced from PS roots and is amenable to long-term storage at room temperature without loss of anti-HIV-1 activity. This indicates the possibility to use PS-polyphenolic drugs as anti-HIV-1 therapy in resource limited settings. Of note, PS is a medicinal plant habituated in South Africa. Hence, it is highly likely that there will be a high level of compliance towards a PS-based antiviral therapeutic regimen in this region, which is strongly affected by the AIDS pandemic.

## Conclusions

Here we demonstrate that an aqueous extract from roots of *Pelargonium sidoides* plants contains robust and potent anti-HIV-1 activity. PS extract prevents HIV-1 particles from attaching to host cells and displays a novel mode-of-action different from other HIV-1 entry inhibitors. Chemical analysis indicates that anti-HIV-1 activity is mediated by the concerted action of multiple polyphenolic compounds which can be separated from components of the extract with higher cytotoxicity by adsorption to polyvinylpyrrolidone.

Based on its potent anti-HIV activity and its extensively studied safety profile, we conclude that PS extract represents a promising lead candidate for the development of a herbal medicine for HIV-1 treatment. The novel mode of anti-HIV activity suggests that PS extract may be useful for complementation of other anti-HIV-1 agents in the therapy of HIV-infected individuals and in protection against HIV-1 infection. Finally, our study demonstrates the amenability of PS extract for experimental analyses, suggesting the use of PS extract as a research tool to advance the development of new anti-HIV agents that protect potential HIV-1 target cells from virus exposure in humans.

## Supporting Information

Materials and Methods S1 Assays for testing of inhibition of HIV-1 infection. Quantification of HIV-1 DNA- and RNA-levels. Chromatographic separation of PS extracts. Ultrahigh resolution mass spectrometry.
(DOCX)

Figure S1 Inhibition of HIV-1 infection by different preparations of PS root extracts, including the commercial herbal medicine EPs® 7630. PS extract from plants were prepared from dry or fresh PS roots as described in the main text (Materials and Methods). For analysis of anti-HIV activity of the commercial herbal medicine Umckaloabo/EPs®7630 (purchased from a local pharmacist), aqueous samples were prepared by removing ethanol in the commercial formulation by evaporation in an Eppendorf Vacuum Concentrator and restoration of the original sample volume with ddH_2_0. Anti-HIV-1 activity was evaluated in LC5-RIC cultures exposed to HIV1_LAI_. Each extract concentration was tested in triplicate. Fluorescent signal intensities of treated cultures were normalized to those of untreated cultures assayed in the same plate (100% infection). Mean values (columns) and standard deviation of the mean are indicated for each extract dilution.
(DOCX)

Figure S2 **ICR/FT-MS analysis of crude PS extract and a PS-derived polyphenolic (PSPP) fraction with polyvinylpyrrolidone-adsorbing compounds confirms enrichment of polyphenols in the PSPP fraction.** 12 Tesla ICR/FT-MS (Ion Cyclotron Resonance/Fourier Transformation -Mass Spectrometry) measurements in negative electrospray ionization mode were conducted of crude PS extract (A) and PSPP (B) to describe their compositional space. Hundreds of assigned elemental formulas are projected in the van Krevelen diagrams (A, B, C). High H/C ratio indicates relative high aliphaticity and low aromaticity, whereas high O/C ratio relates to oxygenated compounds; the bubble size is proportional to the signal intensities in (A) PS extract, (B) the PSPP fraction and (C) to the signal increase in PVPP relative to PS extracts (ratio of intensities PSPP/PS). Crude Pelargonium extracts possesses a high diversity of small molecule components (<1kDa) within a wide domain of polarity and structural characteristics. The PSPP fraction showed an enrichment of polyphenolic compounds in the domain (H/C 0.5-1; O/C 0.4-0.6, violet circle) (B and C); most aliphatic lipid structures (H/C 1.5-2 O/C 0-0.4) are eliminated in PSPP fraction.
(DOCX)
