# Supplementary material for: The Root Extract of the Medicinal Plant Pelargonium sidoides Is a Potent HIV-1 Attachment Inhibitor
Source: PLoS One. 2014 Jan 29;9(1):e87487. doi: 10.1371/journal.pone.0087487 (PMC3906173; doi:10.1371/journal.pone.0087487)
Supplement: Materials and Methods S1 — Assays for testing of inhibition of HIV-1 infection. Quantification of HIV-1 DNA- and RNA-levels. Chromatographic separation of PS extracts. Ultrahigh resolution mass spectrometry. (DOCX) [file pone.0087487.s001.docx]

**SUPPORTING INFORMATION**

**The root extract of the medicinal plant *Pelargonium sidoides* is a potent HIV-1 attachment inhibitor**

Markus HELFER^1a^, Herwig KOPPENSTEINER^1a^, Martha SCHNEIDER^1a^, Stephanie REBENSBURG^1a^, Sara FORCISI^1b^, Constanze MÜLLER^1b^, Philippe SCHMITT-KOPPLIN^1b^, Michael SCHINDLER^1a^ and Ruth BRACK-WERNER^1a^

^1^Helmholtz Zentrum München - German Research Center for Environmental Health, Ingolstädter Landstrasse 1, D-85764 Neuherberg, Germany: ^1a^Institute of Virology, ^1b^Research Unit Analytical BioGeoChemistry

**Supplementary Materials and Methods**

**Assays for testing of inhibition of HIV-1 infection**

Anti-HIV-1-testing with LC5-RIC cells was performed with the EASY-HIT technology as described in [[1](#_ENREF_1)], using on average 2.5 ng p24 per 2 x 10^4^ cells for infection. Since PS extracts decreased the Tat- and Rev-dependent fluorescent reporter signal (step 1) and infectious virus titers (step 2) with similar efficiencies in this assay (see Fig. 1A and Results), all subsequent LC5-RIC assays used the fluorescent reporter signal intensity as read-out for HIV-1 infection.

For testing of anti-HIV-1 activity in PBMCs, thawed PBMCs were seeded at a density of 1 x 10^6^ cells/ml in 75-cm^2^ flasks and cultured in RPMI-medium containing 10%FCS and 20U/ml hIL-2 (Roche) for 3 days at 37°C. For infection, cells were harvested and dispersed into 96-well plates in IL-2 supplemented medium at a density of 1 x 10^5^ cells in 60 µl medium per well, cultured for 24h and exposed to HIV-1 (25 ng p24 /10^5^ cells) in the presence or absence of serial dilutions of inhibitor compounds (total volume of culture supernatants 120 µl per well). Titers of infectious virus produced by PBMCs were determined 48 h after virus addition, using 20 µl of PBMC- culture supernatant and LC5-RIC cells.

For testing of anti-HIV-1 activity in primary human macrophages, cells were exposed to R5-tropic HIV-1NL4-3 IRES-eGFP (200 ng p24 per 10^5^ cells) and treated with the indicated concentrations of PS extracts. Cells were harvested 7 days post infection and the number of GFP-positive cells was determined by standard fluorescence-activated cell sorting (FACS) measurements, essentially as described in [[2](#_ENREF_2)].

For *pre-incubation assays*, serial dilutions of inhibitors in culture medium (PS extracts or reference inhibitors) were dispensed in three 96-well plates (triplicate wells per inhibitor dilution; total volume 100 µl per well). 1 x 10^4^ LC5-RIC cells in 100 µl medium per well were seeded in 3 other plates and cultured at 37°C for 24h before exposure to virus. For virus pre-incubation, virus inoculum (2.5 ng p24 in 120 µl) was added to the inhibitors, the plate incubated for 4 h at 37°C, and the entire pre-incubated virus/inhibitor mixture transferred to a plate with LC5-RIC cells. For cell pre-incubation, the serial dilutions of inhibitors were transferred to a plate with LC5-RIC cells and the plate incubated for 4 h at 37°C before adding the virus inoculum. For simultaneous exposure to virus and inhibitors (i.e. standard EASY-HIT conditions), compound solution followed by virus inoculum was added to each well with LC5-RIC cells. HIV-1 infection was monitored as described ([[1](#_ENREF_1)] and see above).

*Time-of-addition (TOA) assays* were performed as described in [[1](#_ENREF_1)], using inhibitor concentrations > 5 x EC_50_.

**Quantification of HIV-1 DNA- and RNA-levels**

To determine HIV-1 proviral DNA-copies and viral RNA-levels, PBMCs (DNA) or LC5-CD4 (RNA) cells were seeded in 6-well plates 24 hours prior to infection. Cells were pretreated with PS/PSPP before exposure to HIV-1_LAI_. For RNA quantification, cells were co-treated with 50 nM Efavirenz, to prevent viral RNA turn-over by the reverse transcriptase. After 24 (for DNA) and 4 hours (for RNA) of virus exposure, input-virus was removed by extensive washing and nucleic acids were isolated using the DNA Mini and the RNeasy kit (QIAGEN) according to the manufacturer's manuals.

HIV-1 DNA absolute quantification and RNA relative quantification was determined by qPCR using the LightCycler480 Software (Roche, Mannheim, Germany). For absolute quantification of HIV-1 DNA standard curves were generated by PCR reactions with serial dilutions of genomic DNA from TH4-7-5 cells, which contain a single copy of the HIV-1 provirus [[3](#_ENREF_3)]. The external TH4-7-5 standard was used as reference for determine the HIV-1 DNA copy numbers per cellular equivalent in the PBMC samples. Primer sequences for

the HIV-1 *gag*-*pol* junction and the housekeeping gene ß-globin and conditions for amplification are described in [[4](#_ENREF_4)]. Data are expressed as percent of HIV-1 DNA copy numbers per cell of treated samples relative to untreated samples (100%). For RNA quantification, total RNA from LC5-CD4 was reversely transcribed with random hexamers using the Superscript®III First-Strand Synthesis System (Life Technologies), according to the manufacturer`s protocol. For qPCR primers were used as following: HIV-fw 5’– CCA GTC ACA CCT CAG GTA CCT TTA AGA CC – 3’ and HIV-rev 5’- GTG TGT GGT AGA TCC ACA GAT CAA GG -3’. For normalization of the data, RNA polymerase was used as reference gene and amplified in parallel with the primers PRIIs (5’- GCA CCA CGT CCA ATG ACA -3’) and RPIIas (5’- GTG CGG CTG CTT CCA TAA –3’). A standard LightCycler protocol was used and data was analysed with the second derivative maximum method. Relative expression ratio was calculated with the 2^-ΔΔCT^ method described [[5](#_ENREF_5)]. Data is expressed as fold expression of HIV-1 RNA in infected/treated samples compared to uninfected/untreated samples.

**Chromatographic separation of PS extracts.**

PS extracts were pre-treated for UPLC-MS fractionation by Solid Phase Extraction (SPE). C8 SPE-column (Agilent Bond Elute) was equilibrated by washing first with MeOH and then with 2% formic acid in water (FA). 100 µl of PS extract (40 mg/ml) was loaded onto the equilibrated column, the column washed with FA and eluted with MeOH. The residue was reconstituted in 100 µl of H_2_O/MeOH: 8/2 and transferred in UPLC-MS certified vials. UPLC fractionation of the eluate obtained by SPE was performed with an Acquity UPLC System (Waters) coupled to a maXis Qq-TOF mass spectrometer (Bruker Daltonics, Bremen, Germany). The chromatographic separation was achieved on a Kinetex Phenyl PFP UPLC column (2.1 x 150mm, 1.7 µm,), Phenomenex. The gradient elution buffers were A (H_2_O-MeOH: 95/5 containing 0.1% FA) and B (Methanol) with a flow rate of 0.300 ml/min.

The column oven was set to 60°C and the sample manager temperature at 15 ºC. The injection volume was 10µl and the PS extract was injected four times. The spectra were acquired in continuum and centroid modes within a range of 50-1000 m/z with a rolling average value of 2 and 1.0 Hz spectra scanning rate. Fractions were collected by NanoMate fraction collector (TriVersa) in a 96-well plate. The fractions were freeze-dried and re-constituted in ddH_2_0 for anti-HIV-1 testing and in MeOH/H_2_O:8/2 (dilution factor 1000) for ICR/FT-MS analysis.

**Table. UPLC gradient applied for separation**

| **Time** | **Flow (ml/min)** | **Gradient elution buffer A (%)** | **Gradient elution buffer B (%)** |
| --- | --- | --- | --- |
| 0 | 0.3 | 100 | 0 |
| 7 | 0.3 | 50 | 50 |
| 8 | 0.3 | 0 | 100 |
| 8.50 | 0.3 | 0 | 100 |
| 8.51 | 0.3 | 100 | 0 |
| 10.00 | 0.3 | 100 | 0 |

**Ultrahigh resolution mass spectrometry**

Ultrahigh-resolution mass spectra were acquired on a Bruker (Bremen, Germany) solariX ion cyclotron resonance Fourier transform MS equipped with a 12 Tesla superconducting magnet and an Apollo II source [[6](#_ENREF_6)]. The samples were infused via automatic Gilson Sampler.

The MS sampling electrodes, consisting of the spray shield and the metal cap mounted on the high-pressure side of the transfer capillary, were set at 3500 V and 4000V, respectively. A drying-gas flow of 2L/min at 200C was used. The spectra were acquired with a time domain of 2MW over a mass range of 122.89 - 1000 m/z, 300 scans were accumulated for each spectrum of each fraction, 450 for crude and PVPP treated PS extracts. Spectra were externally calibrated first on clusters of arginine (2.5 mg/l in methanol) and internally calibrated on a Fatty acid reference list in negative; calibration errors in the relevant mass range were always below 100 ppb. The spectra exported from DataAnalysis were aligned through in-house software; the data were stored in a matrix by aligning all the peaks within an error of 1 ppm. Masses of an intensity larger than 4.5 x 10^6^ and S/N > 4 were submitted to MassTRIX (www.masstrix.org) [[7](#_ENREF_7)] with a max. error of 1 ppm. This web server assigns to each m/z value potential annotations from KEGG (http://www.genome.jp/kegg/) and LipidMaps (http://www.lipidmaps.org/).

The elemental compositions of the detected masses (m/z-values) of both extracts were calculated by mass difference networking considering H,C,O,N,S and P (maximal error<0.1ppm) [[8](#_ENREF_8)].

Statistical analysis was conducted using SIMCA 9.0 (Umetrics, Sweden). In the PLS-DA model strongly active fractions separated from non-active fractions in the first component (R² (t_1_)0.736, Q²(t_1_) 0.46). The model was validated by a permutation testing using 200 iterations.

**References**

1. Kremb S, Helfer M, Heller W, Hoffmann D, Wolff H, et al. (2010) EASY-HIT: HIV full-replication technology for broad discovery of multiple classes of HIV inhibitors. Antimicrobial agents and chemotherapy 54: 5257-5268.

2. Schindler M, Rajan D, Banning C, Wimmer P, Koppensteiner H, et al. (2010) Vpu serine 52 dependent counteraction of tetherin is required for HIV-1 replication in macrophages, but not in ex vivo human lymphoid tissue. Retrovirology 7: 1.

3. Neumann M, Felber BK, Kleinschmidt A, Froese B, Erfle V, et al. (1995) Restriction of human immunodeficiency virus type 1 production in a human astrocytoma cell line is associated with a cellular block in Rev function. Journal of virology 69: 2159-2167.

4. Kabamba-Mukadi B, Henrivaux P, Ruelle J, Delferrière N, Bodéus M, et al. (2005) Human immunodeficiency virus type 1 (HIV-1) proviral DNA load in purified CD4+ cells by LightCycler® Real-time PCR. BMC infectious diseases 5: 15.

5. Livak KJ, Schmittgen TD (2001) Analysis of Relative Gene Expression Data Using Real-Time Quantitative PCR and the 2< sup>− ΔΔCT</sup> Method. methods 25: 402-408.

6. Hertkorn N, Frommberger M, Witt M, Koch B, Schmitt-Kopplin P, et al. (2008) Natural organic matter and the event horizon of mass spectrometry. Analytical chemistry 80: 8908-8919.

7. Suhre K, Schmitt-Kopplin P (2008) MassTRIX: mass translator into pathways. Nucleic Acids Res 36: W481-484.

8. Tziotis D, Hertkorn N, Schmitt-Kopplin P (2011) Kendrick-analogous network visualisation of ion cyclotron resonance Fourier transform mass spectra: improved options for the assignment of elemental compositions and the classification of organic molecular complexity. Eur J Mass Spectrom (Chichester, Eng) 17: 415-421.
