# Supplementary material for: The Root Extract of the Medicinal Plant Pelargonium sidoides Is a Potent HIV-1 Attachment Inhibitor
Source: PLoS One. 2014 Jan 29;9(1):e87487. doi: 10.1371/journal.pone.0087487 (PMC3906173; doi:10.1371/journal.pone.0087487)
Supplement: Figure S1 — Inhibition of HIV-1 infection by different preparations of PS root extracts, including the commercial herbal medicine EPs® 7630. PS extract from plants were prepared from dry or fresh PS roots as described in the main text (Materials and Methods). For analysis of anti-HIV activity of the commercial herbal medicine Umckaloabo/EPs®7630 (purchased from a local pharmacist), aqueous samples were prepared by removing ethanol in the commercial formulation by evaporation in an Eppendorf Vacuum Concentrator and restoration of the original sample volume with ddH20. Anti-HIV-1 activity was evaluated in LC5-RIC cultures exposed to HIV1LAI. Each extract concentration was tested in triplicate. Fluorescent signal intensities of treated cultures were normalized to those of untreated cultures assayed in the same plate (100% infection). Mean values (columns) and standard deviation of the mean are indicated for each extract dilution. (DOCX) [file pone.0087487.s002.docx]

**Supplementary Figure 1.**

**
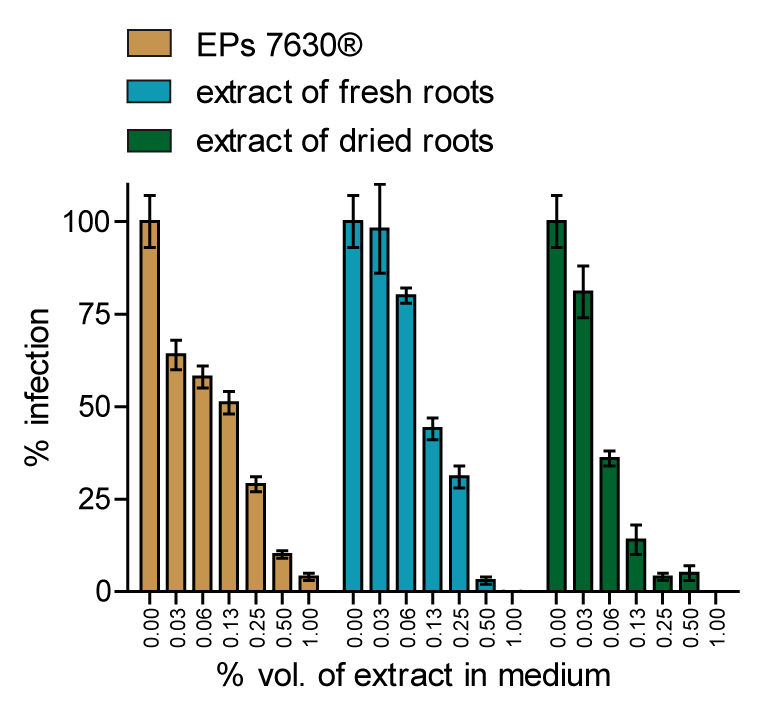
**

**Inhibition of HIV-1 infection by different preparations of PS root extracts, including the commercial herbal medicine EPs^®^ 7630**. PS extract from plants were prepared from dry or fresh PS roots as described in the main text (Materials and Methods). For analysis of anti-HIV activity of the commercial herbal medicine Umckaloabo/EPs^®^7630 (purchased from a local pharmacist), aqueous samples were prepared by removing ethanol in the commercial formulation by evaporation in an Eppendorf Vacuum Concentrator and restoration of the original sample volume with ddH_2_0.

Anti-HIV-1 activity was evaluated in LC5-RIC cultures exposed to HIV1_LAI_. Each extract concentration was tested in triplicate. Fluorescent signal intensities of treated cultures were normalized to those of untreated cultures assayed in the same plate (100 % infection). Mean values (columns) and standard deviation of the mean are indicated for each extract dilution.
