# Supplementary material for: The Root Extract of the Medicinal Plant Pelargonium sidoides Is a Potent HIV-1 Attachment Inhibitor
Source: PLoS One. 2014 Jan 29;9(1):e87487. doi: 10.1371/journal.pone.0087487 (PMC3906173; doi:10.1371/journal.pone.0087487)
Supplement: Figure S2 — ICR/FT-MS analysis of crude PS extract and a PS-derived polyphenolic (PSPP) fraction with polyvinylpyrrolidone-adsorbing compounds confirms enrichment of polyphenols in the PSPP fraction. 12 Tesla ICR/FT-MS (Ion Cyclotron Resonance/Fourier Transformation -Mass Spectrometry) measurements in negative electrospray ionization mode were conducted of crude PS extract (A) and PSPP (B) to describe their compositional space. Hundreds of assigned elemental formulas are projected in the van Krevelen diagrams (A, B, C). High H/C ratio indicates relative high aliphaticity and low aromaticity, whereas high O/C ratio relates to oxygenated compounds; the bubble size is proportional to the signal intensities in (A) PS extract, (B) the PSPP fraction and (C) to the signal increase in PVPP relative to PS extracts (ratio of intensities PSPP/PS). Crude Pelargonium extracts possesses a high diversity of small molecule components (<1kDa) within a wide domain of polarity and structural characteristics. The PSPP fraction showed an enrichment of polyphenolic compounds in the domain (H/C 0.5-1; O/C 0.4-0.6, violet circle) (B and C); most aliphatic lipid structures (H/C 1.5-2 O/C 0-0.4) are eliminated in PSPP fraction. (DOCX) [file pone.0087487.s003.docx]

**Supplementary Figure 2.**

**
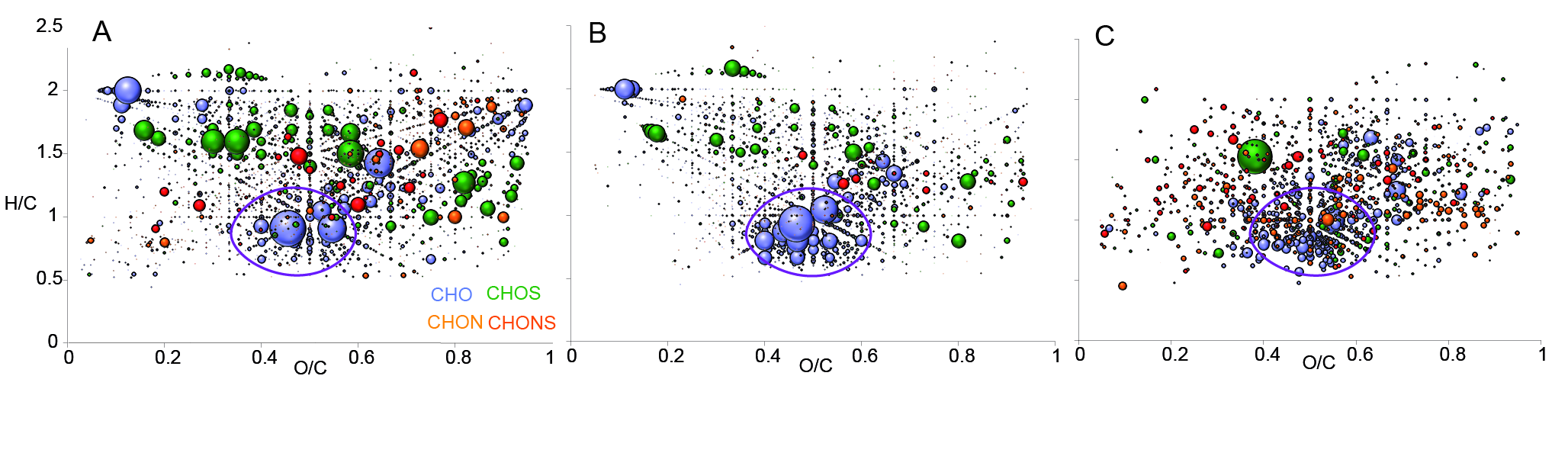
**

**ICR/FT-MS analysis of crude PS extract and a PS-derived polyphenolic (PSPP) fraction with polyvinylpyrrolidone-adsorbing compounds confirms enrichment of poylphenols in the PSPP fraction.**

12 Tesla ICR/FT-MS (Ion Cyclotron Resonance/Fourier Transformation -Mass Spectrometry) measurements in negative electrospray ionization mode were conducted of crude PS extract (A) and PSPP (B) to describe their compositional space. Hundreds of assigned elemental formulas are projected in the van Krevelen diagrams (A, B, C). High H/C ratio indicates relative high aliphaticity and low aromaticity, whereas high O/C ratio relates to oxygenated compounds; the bubble size is proportional to the signal intensities in (A) PS extract, (B) the PSPP fraction and (C) to the signal increase in PVPP relative to PS extracts (ratio of intensities PSPP/PS). Crude Pelargonium extracts possesses a high diversity of small molecule components (<1kDa) within a wide domain of polarity and structural characteristics. The PSPP fraction showed an enrichment of polyphenolic compounds in the domain (H/C 0.5-1; O/C 0.4-0.6, violet circle) (B and C); most aliphatic lipid structures (H/C 1.5-2 O/C 0-0.4) are eliminated in PSPP fraction.
